# Supplementary material for: Combinations of action observation and motor imagery on golf putting’s performance
Source: PeerJ. 2022 May 11;10:e13432. doi: 10.7717/peerj.13432 (PMC9107300; doi:10.7717/peerj.13432)
Supplement: Supplemental Information 2 [file peerj-10-13432-s002.docx]

Code Book

Project title: Combinations of action observation and motor imagery on golf putting’s performance

# Column 1: number of participants (1=group; number=participant in each group)

# Column 2: group (1= simultaneous action observation combined with motor imagery (S-AOMI); 2= alternate AOMI (A-AOMI) which performs action observation first, then motor imagery; 3= synthesized action observation and motor imagery (A-S-AOMI) which perform A-AOMI, then S-AOMI; 4= control

# Column 3: gender (males=1; females=2)

# Column 4: age

# Column 5-12: (MIQ 1-8)

# Column 13: pretest

# Column 14: posttest

# Column 15: retention #1

# Column 16: retention #2

# Column 17: kinesthetic ability of MIQ

# Column 18: visual ability of MIQ

# Column 19-26: intervention questionnaire 1-7
