# Supplementary material for: Combinations of action observation and motor imagery on golf putting’s performance
Source: PeerJ. 2022 May 11;10:e13432. doi: 10.7717/peerj.13432 (PMC9107300; doi:10.7717/peerj.13432)
Supplement: Supplemental Information 3 [file peerj-10-13432-s003.docx]

Imagery scripts for the PETTLEP intervention in golf putting

Imagine you are situated at the sport psychology lab of the National Taipei University. Eight LED light bulbs with 450 lumens project the light on the space of the lab. The temperature is about 25 centigrade, so it is mildly comfortable (P, physical). There are tables, chairs, a bookshelf, a golf-putting turf, golf clubs, balls, and an experimenter and assistant in the lab. This place is where you are about to perform golf putting (E, environment). You are standing at the artificial turf and firmly hold a golf club ready to perform golf putting to a hole that is 180 centimeters away. You adjust your body as a golf instructor teaches you. You look at the golf artificial turf and hole. Image how much force you are about to put the golf ball (T, task). While putting, you are aiming at the hole and forming a motor program in your mind, so you put it as real-time as it should be (T, time). The goal of this putting is to improve your golf skill. So what you should do is do it as better as you can (L, learning). You feel as calm and relaxed as your experience that used to be. This psychological state helps you in total concentration and control (E, emotion). While putting, you feel the sensation of the force to put the ball. You rotate your trunk to generate an appropriate force. Finally, you see the golf ball going the artificial route directly into the hole (P, perspective).
